# Case of Traumatic Tetanus, in which Ice was Beneficially Applied to the Spine

**Published:** 1846-04

**Authors:** John E. McGirr

**Affiliations:** Hollidaysburg, Blair Co., Pa.


### MEDICAL EXAMINER,

AND

### RECORD OF MEDICAL SCIENCE.

NEW SERIES.—No. XVI.—APRIL, 1846.

### ORIGINAL COMMUNICATIONS.

### *Case of Traumatic Tetanus, in which Ice was beneficially applied to the Spine.* By John E. McGirr, M. D.

Miss Margaret C——, aged 20, of plethoric habit, applied on the 28th January to a dentist to have a tooth extracted—a second molar, in the lower jaw, on the right side. The dentist broke the tooth; and after several ineffectual efforts to remove the stump, applied some caustic to destroy the nerve, (supposed to be nitric acid,) and sent her home.

On Monday night, February 2d, at 9 o'clock, five days after the tooth was broken, tetanus came on. She had suffered excessive pain in the jaw, day and night, from the time the caustic was applied. Being summoned immediately after the first spasm, I was, from indisposition, unable to attend, and another physician was called in. As the case grew rapidly worse, the friends fearing a fatal termination, requested me to take charge of it, which I did on Wednesday morning. I found her in the following condition.

From one to four spasms occurring every hour. At 2 o'clock, P. M., the worst occurred; and from that time until the turn of the night they were very frequent and severe. The jaws were tightly locked; opisthotonos was severe; and there was pain and retraction at the epigastrium, returning every twenty or twenty-five minutes, followed by excessively violent paroxysms, —three generally succeeding each other within a minute's time.

The temporal and masseter muscles were the seat of very great pain. The head was thrown back, and every muscle was in a state of rigid contraction. She answered, by signs, that there were severe pains shooting from the sternum to the spine. Swallowing was performed with the greatest difficulty; and twice to-day the very effort produced spasms. The belly was very hard and painful on being pressed; and when pressed upon, muscular twitchings agitated the entire body. These twitchings were constantly present between the spasms, and the muscles of the entire body were affected during the paroxysms. The muscles of the head, neck, throat, chest, extremities, hands and fingers, were painfully contracted. The eyelids were drawn down tightly over the eyes; the patient could not move them; and when I raised them, which I found it difficult to do, the eyes were turned up, insensible to light, and the pupils largely dilated. The countenance was so much distorted, that I would not have recognized her as one with whom I was well acquainted. The pulse was wiry and beating 130. There was suppression of urine for 24 hours.

I ordered a blister to be applied from the temple to the symphysis of the inferior maxillary bone, (to be dressed with morphia and mercurial ointment, and a warm poultice over the dressings;) and bags of ice to the whole tract of the spine. When the ice was applied, a spasm immediately occurred, and it was removed; but when the patient recovered her consciousness, (which always returned between the spasms,) she made her attendants understand that she wished the ice replaced, which was done. One grain of morphia was ordered every hour, until stertorous breathing was induced, and perfect quiet was enjoined.

Feb. 5th, 9 o'clock, A. M. The most part of last night was passed badly,—the spasms occurring about every two hours, until four o'clock this morning, when an intermission occurred, and the sufferer fell into a quiet slumber which lasted four hours. She awoke without a spasm, motioned for a drink—which was given to her, and swallowed with much less difficulty than yesterday. The bowels were freely moved by a purgative. The eyes and eyelids were immoveable. The pulse 80, and full; and the skin moist. Suppression of urine relieved.

2, P. M. Has had three spasms since morning; and while I was in the house she vomited about a teacupful of a dark green matter. The vomiting was followed by three spasms in succession. Morphia resumed.

8, P. M. No spasm since last visit. She had three while I was in the house. The pulse small and corded; breathing oppressed. Ordered morphia in small doses, and wine freely.

Fearing too great exhaustion, the ice was removed from the spine, and the most perfect rest enjoined, as the running of a wagon on the road above the house produced a spasm.

6th. 7, A. M. Spent the early part of last night without a spasm; the latter was passed rather restlessly, and with two spasms, which were much lighter than usual. Vomited once, which was followed by a spasm. She was sleeping when I saw her; the breathing stertorous. Pulse 85, and full. The jaws are open about a quarter of an inch, and slightly moveable. Ordered a blister to the spine, in consequence of some remaining pain, to be dressed with mercurial ointment, and a warm poultice over the dressings; the morphia to be resumed after the stertorous breathing passes off, in doses of a quarter of a grain every two hours.

1, P. M. No spasm since morning; has slept soundly. At 2 o'clock, before I left the house, she had three slight spasms. Morphia continued, with two drops of croton oil, to be repeated if necessary. Vomited after taking the oil, which was then repeated, dose two drops. The vomiting produced no spasms. Feels a tingling sensation passing up from the extremities.

6, P. M. Has opened her eyes and spoken for the first time since her attack. The jaws are relaxed and moveable. The blister drew well. Complains still of pain shooting from the sternum to the spine. She says that, "until the ice was applied, this pain was terrible." Is hungry; has eaten some cracker grated in chocolate; has used wine, beef-tea, and arrow-root jelly, to support her strength. No muscular twitchings. She breathes and swallows easily; says her heart jumps a good deal yet. Her eyes are open, but she cannot see. Morphia in small doses until sleep is induced.

7th. 8, A. M. Rested well last night until one o'clock, when she had a slight spasm; moving her to the bed pan produced two others. She speaks distinctly. The jaws are relaxed; the eyes open, but she can see nothing. Pulse weak. Ordered wine freely; and as the bowels had not yet been moved, one drop of croton oil was given. Complains of pain in the jaws; a few doses of morphia ordered, quarter of a grain each.

4, P. M. Escaped the 2 o'clock spasm to-day for the first time. Slept from the morning visit. The bowels not yet moved. Ordered, of a mixture consisting of equal parts of the spt. turpentine and castor oil, two table-spoonfuls in a cup of starch-water, to be given as an injection every hour.

8th. Has had but one spasm since yesterday at 4 o'clock, and that was produced by giving an injection. Three injections were given, by which two copious evacuations were procured. The catamenia came on with the alvine evacuations; and it is to be noted, that her regular and healthy menstruation had ceased naturally two days before the tooth was broken. She complains of pain in the temporal and masseter muscles; talks distinctly, and lies quietly.

5, P. M. Had one slight spasm at 1 o'clock. Has been moved without inconvenience. Sleeps well. Can now distinguish light from darkness. The menstrual discharge continues. Morphia to be discontinued, as it sickens; spt. nit. æther to be given freely, and the morphia to be resumed should spasms threaten. Pulse 80, and full; the jaws open; tongue benumbed; she says it felt very much swollen in the early part of the disease, and that she could always tell when the spasms were approaching by the gradual coming on of numbness of the tongue; and that the numbness would begin at the root, and when it had reached the point, the spasms would attack her.

9th. 10, A. M. Has had no spasm since yesterday; one very offensive evacuation from the bowels; the menses decreasing. She raved very much last night, but was thrown into a very sound sleep by three tea-spoonfuls of weak laudanum. Has slight pain in the jaws and head; the eyesight improving; pulse good.

I have taken notes up to the 14th February, but as nothing of any importance occurred, it is unnecessary to continue them in detail. Suffice it to say, that on the 11th the menses stopped; on the same day a quantity of blood and mucus passed from the bowels, after two or three very severe pains, and about an hour's sickness; and on the same day the eyesight was completely restored. On the 12th there was a threatened relapse, in consequence of catching cold; this was easily arrested, and under generous diet, and great care from her nurse, she rapidly convalesced from this time, though months may be required before she recovers her wonted strength and spirits.

REMARKS.

There are some things in this case worth noting. The loss of sight was something that I had never read of as occurring in this disease. The spasms, from the first day I saw her, affected the fingers, hands, face, throat, breast, back, abdomen and legs; they were so severe, that four men were required to hold her upon the bed. There was abdominal hardness; severe opisthotonos; and pain from the sternum to the spine. To me, having seen several and always fatal cases of this kind, the action of the ice was perfectly astonishing; it produced almost immediate relaxation of tonic rigidity, and a remission of spasmodic action beyond my most sanguine expectations. Had the ice been applied when the patient was first attacked, I believe she would have been spared a great deal of suffering. She says that when the ice was applied to the spine, it occasioned a delightful feeling of relief, as if an enormous weight was removed from her breast; and that when the spasms came on during the time the ice was applied, they caused her but trifling pain compared with what she had previously endured.

*Hollidaysburg*, Blair Co., Pa., Feb. 28th, 1846.

